# Optimizing the production and efficacy of antimicrobial bioactive compounds from *Streptomyces kanamyceticus* in combating multi-drug-resistant pathogens

**DOI:** 10.3389/fcimb.2024.1500440

**Published:** 2025-01-06

**Authors:** Zifang Shang, Vipasha Sharma, Liu Pai, Tarun Kumar, Sandip Patil

**Affiliations:** ^1^ Guangdong Engineering Technological Research Centre of Clinical Molecular Diagnosis and Antibody Drugs, Meizhou Academy of Medical Sciences, Meizhou People’s Hospital (Huangtang Hospital), Meizhou, China; ^2^ Department of Biotechnology, University Institute of Biotechnology, Chandigarh University, Mohali, Punjab, India; ^3^ Department of Haematology and Oncology, Shenzhen Children’s Hospital, Shenzhen, China; ^4^ Mkelly Biotech Pvt Ltd., Mohali, Punjab, India; ^5^ Paediatric Research Institute, Shenzhen Children’s Hospital, Shenzhen, China

**Keywords:** *Streptomyces kanamyceticus*, antimicrobial activity, bioactive compounds, optimization, antibiotic resistance

## Abstract

**Background:**

The rise of antibiotic-resistant pathogens has intensified the search for novel antimicrobial agents. This study aimed to isolate *Streptomyces kanamyceticus* from local soil samples and evaluate its antimicrobial properties, along with optimizing the production of bioactive compounds.

**Methods:**

Soil samples were collected from local regions, processed, and analysed for Streptomyces strains isolation using morphological characteristics and molecular identification through 16S rRNA gene PCR assay. Antimicrobial activity was assessed against *Escherichia coli*, *Staphylococcus aureus*, *Bacillus subtilis*, and *Candida albicans* using the double-layer method, while Minimum Inhibitory Concentration (MIC) values were determined. The extracted compounds underwent Fourier Transform Infrared Spectroscopy (FTIR) analysis for functional group identification. Optimization of bioactive compound production was performed using a Central Composite Design (CCD) coupled with Partial Least Squares Regression (PLSR).

**Results:**

A total of 25 distinct *Streptomyces* strains were isolated, with seven confirmed as *S. kanamyceticus*. These strains exhibited antimicrobial activity, with inhibition zones reaching 30 mm and MIC values between 20 and 70 µg/mL. The extraction yielded 150-200 mL of bioactive compounds. Optimization studies revealed that a medium containing 10 g/L glucose and 10 g/L glycine max meal maximized antibiotic production.

**Conclusion:**

This study confirmed that *S. kanamyceticus* is a promising source of novel antibiotics. The combination of microbial isolation, antimicrobial testing, and statistical optimization successfully enhanced the production of bioactive compounds, contributing to the search for effective antimicrobial agents against resistant pathogens.

## 1 Introduction

The global rise of multidrug-resistant (MDR) pathogens represents a critical public health challenge, with serious implications for healthcare systems worldwide (Kumar et al., 2024). MDR infections have become a major cause of morbidity and mortality, with nearly 700,000 deaths annually, a figure projected to rise to 10 million by 2050 if current trends persist (Tang et al., 2023). This crisis is exacerbated by the indiscriminate use of antibiotics, particularly broad-spectrum antibiotics like cephalosporins, fluoroquinolones, and carbapenems, which have accelerated the emergence of resistant strains (Aslam et al., 2018). About 50% of antibiotics prescribed are either inappropriate or unnecessary, contributing to the rapid development of resistance (Ventola, 2015). The limitations of currently available antibiotics, including penicillin derivatives, tetracyclines, and macrolides, further compound the problem as many first-line treatments are losing efficacy against resistant organisms such as *Escherichia coli*, *Klebsiella pneumoniae*, and *Staphylococcus aureus* (Saxena et al., 2023). This pressing issue highlights the urgent need for novel antimicrobial agents capable of effectively combating MDR pathogens. Despite decades of research, the discovery of new antibiotics has been remarkably slow, with only a handful—such as daptomycin and ceftaroline—introduced in the last 40 years (D’Andrea et al., 2019; Uddin et al., 2021). This stagnation is largely due to the high costs and extended timelines associated with antibiotic development, paired with the limited financial returns from drugs that are typically used only for short durations (Bergkessel et al., 2023). The pharmaceutical industry has largely shifted its focus away from antibiotic development due to the lower profitability of antimicrobial agents. This has resulted in a limited pipeline of novel drugs (Powers, 2004). Furthermore, most recent efforts have yielded only minor improvements on existing therapies, rather than producing innovative breakthrough solutions. The scarcity of new antibiotics has led to an increased reliance on last-resort drugs like colistin and vancomycin (Mondal et al., 2024). Alarmingly, resistance to these vital drugs is also emerging, underscoring the need to explore alternative sources of antimicrobial compounds. Natural products, particularly phytochemicals such as flavonoids, alkaloids, and terpenes, have long been recognized as rich sources of bioactive compounds with therapeutic potential (Riaz et al., 2023). Notably, over 70% of all antibiotics in clinical use, including penicillin and vancomycin, were derived from natural sources. In recent years, research efforts have turned toward underexplored reservoirs such as plant-based compounds and microbial metabolites. Numerous studies have shown the efficacy of phytochemicals like curcumin and berberine in inhibiting bacterial growth, often with lower risks of resistance development compared to synthetic antibiotics (Martens and Demain, 2017). However, despite the promising potential of these natural compounds, large-scale, in-depth research remains limited. Most studies are still in their preliminary stages, and there is a pressing need for more comprehensive investigations on a global scale (Barba-Ostria et al., 2022). One particularly promising source of bioactive compounds is *Streptomyces*, a genus of soil-dwelling bacteria renowned for its ability to produce a wide range of antibiotics, including tetracycline, chloramphenicol, and erythromycin, along with other secondary metabolites like antitumor agents and immunosuppressants (Khan et al., 2023). *Streptomyces* has played a pivotal role in antibiotic discovery, most notably as the source of streptomycin, the first effective treatment for tuberculosis (Quinn et al., 2020). The genus is recognized for producing diverse bioactive compounds with antimicrobial, antifungal, and antitumor properties. Continued exploration of *Streptomyces* species remains essential for the identification of new bioactive compounds (Mazumdar et al., 2023). In this study, we focus on optimizing the production and efficacy of bioactive compounds from *S. kanamyceticus* in combating MDR pathogens. Our objective is to enhance the yield and potency of these compounds using Central Composite Design (CCD) and Partial Least Squares Regression (PLSR) analysis. By doing so, we aim to uncover the potential of *S. kanamyceticus* as a source of novel antimicrobial agents, contributing to the global fight against MDR infections.

## 2 Methods

### 2.1 Chemicals

All chemicals, media components, and standard antibiotics utilized in this study were sourced from Hi-Media Pvt. Ltd. and Sigma-Aldrich Corporation (India). The solvents used throughout the experiments were of analytical or HPLC grade, obtained from SD Fine Chem Limited (India).

### 2.2 Isolation and characterization of *Streptomyces* species

A total of 10 soil samples were systematically collected from the loacal region in February 2023, specifically at Latitude 30.7333° N and Longitude 76.7794° E. Sampling sites were randomly selected from various distances within the area. To ensure diversity in *Streptomyces* species, soil was collected from five distinct points within a 400 m² zone for each habitat. At each point, the top 6 cm of soil was removed using a sterile spatula. Subsequently, 100 to 120 grams of soil from the underlying layer were collected, placed in stomacher sachets, mixed, and homogenized to produce a heterogeneous sample (Mazumdar et al., 2023). All soil samples were collected in sterile containers and stored immediately at 4°C until further processing. To initiate the isolation process, soil samples (1gram) were suspended in sterile saline (9ml), and serial dilutions were spread onto starch-nitrate agar medium, which was supplemented with 50 μg/ml of cycloheximide and 30 μl/ml of nalidixic acid. The agar plates were incubated at 37°C for 5 days. Potentially valuable isolates were identified by selecting grey colonies that secreted pigments. These selected colonies were then streaked onto starch casein nitrate (SCN) agar containing 25 µg/ml cycloheximide and 50 µg/ml rifampicin, with further incubation at 37°C for another 5 days. After incubation, the isolates were characterized based on the International *Streptomyces* Project (ISP) criteria, which included an evaluation of mycelium shape, colour, substrate mycelium, melanin production, and soluble pigment production. Stock cultures of the 20 selected isolates were preserved as spore and mycelial suspensions in 20% glycerol at -20°C for future reference. To confirm the species, morphological assessments were conducted on the 20 isolates. Species confirmation involved performing a PCR assay targeting the 16S rRNA gene using forward primer (5’-AGAGTTTGATCMTGGCTCAG-3’) and reverse primer (5’-TACGGYTACCTTGTTACGACTT-3’) (Chen et al., 2015). The amplified products were visualized through electrophoresis on a 1.8% agarose gel stained with 0.5 μg/ml ethidium bromide. DNA sequencing of the PCR products was carried out by a commercial service provider (Genentech). The resulting sequences were analyzed using the Basic Local Alignment Search Tool (BLAST) on the National Center for Biotechnology Information (NCBI) platform. These sequences were compared with GenBank database sequences (www.ncbi.nlm.nih.gov), and a phylogenetic tree was constructed to determine evolutionary relationships.

### 2.3 Primary screening for antimicrobial activity

To identify the optimal medium for antibiotic production, the antimicrobial activity of pure isolates was evaluated using the double-layer method on various ISP agar media. First, different ISP agar media, including ISP2, ISP3, ISP4, and ISP5, were prepared according to standard protocols (Rajeswari et al., 2014). These media were selected to assess the performance of S*treptomyces* under different nutrient conditions. Streptomyces isolates were then inoculated onto the prepared ISP agar media and incubated for a specified period, typically 7 days, to promote optimal growth and antibiotic production. Subsequently, test organisms, including *Escherichia coli* ATCC 25922 (Gram-negative bacterium), *Staphylococcus aureus* ATCC 25923 (Gram-positive bacterium), *Bacillus subtilis* ATCC 19659 (non-pathogenic Gram-positive bacterium), and *Candida albicans* ATCC 60193 (pathogenic yeast), were used to evaluate antimicrobial activity. The plates were then incubated at 37^°^C for 24 to 48 hours for bacterial strains and at 25^°^C for 48 to 72 hours for *Candida albicans*. Following incubation, antimicrobial activity was assessed by measuring the zones of inhibition around the Actinomycetes colonies. The measurement of these zones (mm) provided an indication of the antimicrobial effectiveness of the isolates. Larger zones of inhibition signified greater antimicrobial activity. The results were analysed to determine which ISP media supported the highest levels of antimicrobial activity. This comparison facilitated the selection of the most effective medium for optimizing antibiotic production.

### 2.4 Extraction method for bioactive compounds from *Streptomyces* spp.

To extract bioactive compounds from *Streptomyces* spp., the culture broth was filtered through sterile cheesecloth to remove mycelial debris The filtrate, typically 100 mL, was transferred to a separatory funnel. To this, an equal volume of diethyl ether (Et2O) was added. The mixture was shaken vigorously for 10 minutes to ensure thorough mixing. Afterwards, the mixture was allowed to settle, and the organic layer, which contained the extracted compounds, was carefully separated and collected. Concurrently, the mycelial mass was homogenized with diethyl ether in a mortar and pestle, using approximately 100 mL of solvent for the homogenization. The resulting homogenate was filtered through a fine mesh to obtain the liquid extract. This liquid was then combined with the organic layer from the culture broth extraction. The combined diethyl ether extracts were concentrated using a rotary evaporator at 40°C to remove the solvent (Ibnouf et al., 2022). The concentrated extract was transferred to a clean glass vial and stored at -20°C until further analysis.

### 2.5 Determination of minimum inhibitory concentration

To determine the MIC of the bioactive compounds extracted from *Streptomyces* spp., the Kirby-Bauer disc diffusion method was utilized. Mueller-Hinton agar plates were prepared for bacterial assays, while Sabouraud Dextrose agar plates were used for fungal assays. The test microorganisms—*Escherichia coli*, *Staphylococcus aureus*, and *Candida albicans*—were cultured to an appropriate turbidity, specifically a 0.5 McFarland standard, which corresponds to approximately 1.5 × 10^8^ CFU/mL. This turbidity standard ensures consistent inoculum density across different assays. The cultures were then evenly spread onto the surface of the agar plates using a sterile spreader to achieve a uniform lawn of growth. Sterile filter paper discs were impregnated with various concentrations of the concentrated extract. Typically, concentrations ranged from 10 µg, 20 µg, 40µg to 100 µg per disc, though specific concentrations may vary depending on the extract’s potency and experimental design. The discs were placed on the inoculated agar plates with sufficient spacing to prevent overlapping inhibition zones. The plates were incubated at 37°C for 24 hours for bacterial strains and at 25°C for 48 to 72 hours for *Candida albicans*. After incubation, the zones of inhibition around the discs were measured in mm (Sharma et al., 2016). The MIC was determined by identifying the lowest concentration of the extract which resulted in a clear zone of inhibition around the disc, indicating effective suppression of microbial growth. This approach provided a quantitative measure of the antimicrobial activity of the extracts against the selected pathogens.

### 2.6 FTIR analysis of diethyl ether extracts from *Streptomyces* SK-2023-2 and SK-2023-4

The diethyl ether extracts of Streptomyces strains SK-2023-2 and SK-2023-4 were analyzed using Fourier Transform Infrared Spectroscopy (FTIR) to identify functional groups and characterize the chemical composition of the bioactive compounds (Chakraborty et al., 2023). 1 mg of each extract was placed on a clean KBr pellet and thoroughly mixed with about 100 mg of dry KBr powder to form a homogeneous mixture. This mixture was then compressed into a transparent pellet using a hydraulic press at a pressure of 10,000 psi for approximately 5 minutes, ensuring that the pellet was uniform and free from air bubbles. FTIR spectra were recorded using an FTIR spectrometer (ReactIR702L) over the range of 4000 cm^-^¹ to 400 cm^-^¹. Each spectrum was acquired at a resolution of 4 cm^-^¹, with 32 scans averaged to enhance the signal-to-noise ratio. The obtained spectra were analyzed to identify characteristic absorption bands corresponding to the functional groups present in the extracts. Peaks were assigned based on comparisons with standard reference spectra and literature values. The spectral data were interpreted to determine the functional groups and possible chemical structures of the bioactive compounds present in the extracts from SK-2023-2 and SK-2023-4.

### 2.7 Statistical optimization of antimicrobial properties

To identify the optimal concentration and interactions of key media components—glucose, glycine max, and CaCO_3_—a systematic investigation was performed using a Central Composite Design (CCD) (Ju et al., 2017). The experimental setup is detailed in **Table 1**, where each factor was evaluated at five different levels: -α, -1, 0, + 1, and +α. Design Expert Software version 13 was utilized to generate a series of 20 experiments, each conducted in triplicate. The average zone of inhibition (mm) was measured as the dependent variable or response. All experiments were prepared and incubated at 28°C and 100 rpm for 5 days.

**Table 1:** Experimental setup for central composite design (CCD) to optimize media components.

Factor	Level -α	Level -1	Level 0	Level +1	Level +α
Glucose (g/L)	5	10	15	20	25
Glycine max (g/L)	5	10	15	20	25
CaCO_3_ (g/L)	0.5	1	1.5	2	2.5

#### 2.7.1 Statistical analysis and modelling

The data obtained from the CCD experiments were subjected to rigorous analysis of variance (ANOVA). A first-order polynomial equation was fitted through multiple regression analysis to develop an empirical model that quantifies the relationship between the measured response and the independent variables. The empirical model is expressed by the following equation:


$$\begin{array}{c} \text{Y}=\text{B}0+\text{B}1{\text{X}}_{1}+\text{B}2{\text{X}}_{2}+\text{B}3{\text{X}}_{3}+\text{B}4\text{X}1{\text{X}}_{2}+\text{B}5\text{X}1{\text{X}}_{3}+\text{B}6{\text{X}}_{2}{\text{X}}_{3}\\ +\text{B}7{\text{X}}_{1}^{2}+\text{B}8{\text{X}}_{2}^{2}+\text{B}9{\text{X}}_{3}^{2} \end{array}$$

Where Y represents the predicted response, and B_0_​ through B_9_​ are the regression coefficients for the respective variables and interaction terms. The independent variables X_1,_ X_2_​, and X_3_ correspond to glucose, glycine max, and CaCO_3_, respectively. The response was further analysed through three-dimensional plots to visualize the effects and interactions of the variables.

#### 2.7.2 Multivariate analysis

A combination of multiple linear regression (MLR) and the white box approach of data modelling was employed, utilizing Partial Least Squares Regression (PLSR) (Kreeger, 2013). This methodology, as discussed in the literature previously aimed to thoroughly understand the correlation between the independent variables (glucose, glycine max, and CaCO_3_) and the zone of inhibition (dependent variable). PLSR is particularly useful in the presence of multicollinearity among predictor variables, allowing the development of both explanatory and predictive models. Additionally, to ensure the robustness of the developed model, a model assessment technique known as leave-one-out cross-validation was applied. This iterative procedure involves creating a regression model for each sample while excluding it as a test case, and then assessing the prediction outcomes. Through these statistical approaches, the complex relationships between the experimental variables and the antifungal properties were extensively explored.

## 3 Results

### 3.1 Identification and characterization of *Streptomyces* spp.

**Figure 1:**
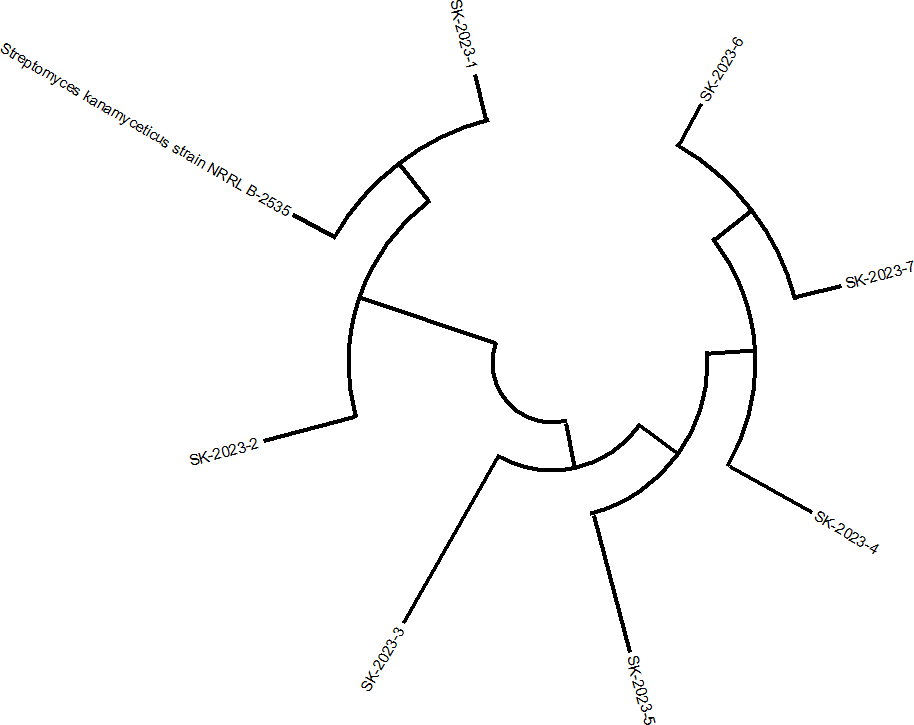
Phylogenetic tree of isolated *Streptomyces* spp. based on 16S rRNA sequencing.

The soil analysis from the site indicated it to be alkaline with a pH of 7.9 and low salinity (EC = 0.15 ds/m). Based on the texture diagram, the soil was classified as sandy loam, characterized by a low clay content (20%), adequate total nitrogen (0.17%), and a moderate level of organic matter (2.98%). The analysis also revealed the presence of exchangeable cations, including potassium (K), magnesium (Mg), aluminium (Al), calcium (Ca), and silicon (Si). Among the mineral elements, oxygen (O), iron (Fe), and silicon (Si) were the most abundant, followed by aluminium, calcium, potassium, and magnesium (**Supplementary Table S1**). From the soil samples collected across the five locations, 25 morphologically distinct presumptive *Streptomyces* strains were successfully isolated. Out of the 25 isolated strains, 7 were identified as *S. kanamyceticus* based on 16S rRNA sequencing, and their phylogenetic relationships were confirmed through a constructed phylogenetic tree (**Figure 1**). These 7 isolates were selected for further study and were designated as SK-2023-1 through SK-2023-7.

### 3.2 Screening of active *S. kanamyceticus* isolates

**Figure 2:**
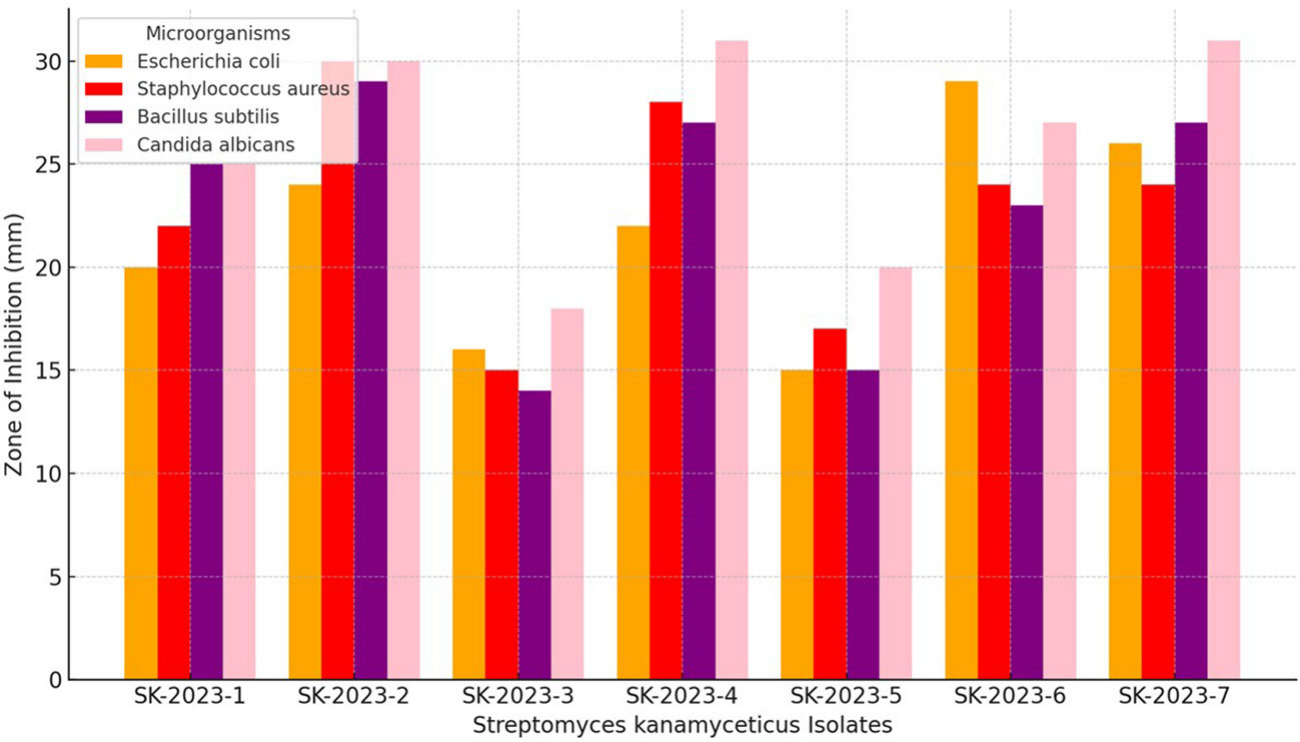
Antimicrobial activity profiles of *S. kanamyceticus* isolates against pathogens.

The primary screening of the *S. kanamyceticus* isolates against *Escherichia coli* ATCC 25922, *Staphylococcus aureus* ATCC 25923, *Bacillus subtilis* ATCC 19659, and *Candida albicans* ATCC 60193 revealed varied levels of antimicrobial activity (**Figure 2**). Isolate SK-2023-1 demonstrated inhibition zones of 20 mm, 22 mm, 25 mm, and 25 mm, respectively, against these microorganisms. SK-2023-2 exhibited the highest inhibition for all tested organisms, with 24 mm against *E. coli*, 30 mm against *S. aureus*, 29 mm against *B. subtilis*, and 30 mm against *C. albicans*. Conversely, SK-2023-3 showed the lowest activity, with inhibition zones of 16 mm, 15 mm, 14 mm, and 18 mm, respectively. SK-2023-4 produced strong inhibition, particularly against *C. albicans* (31 mm) and *S. aureus* (28 mm). The antimicrobial activity of SK-2023-5 was moderate, ranging from 15 mm to 20 mm across all pathogens. SK-2023-6 and SK-2023-7 displayed similar levels of activity, with SK-2023-6 showing higher inhibition against *E. coli* (29 mm), while both exhibited strong inhibition against *C. albicans* (27 mm and 31 mm, respectively). These results indicate significant variability in the antimicrobial efficacy of the different *S. kanamyceticus* isolates.

### 3.3 Extraction and minimum inhibitory concentration

The extraction of bioactive compounds from *Streptomyces* spp. was successfully achieved, yielding approximately 150-200 mL of concentrated extracts suitable for further analysis. The concentrated extracts exhibited distinct colours and viscosities, indicating the presence of various metabolites. Subsequent antibacterial and antifungal activity assays revealed significant antimicrobial potential of the bioactive compounds. The MIC (MIC) and the corresponding Zone of Inhibition were recorded (**Table 2**). *Escherichia coli* ATCC25922 exhibited a MIC ranging from 20 µg/ml to 70 µg/ml, with zone inhibition measurements varying from non-detectable to substantial inhibition (29 mm). Similarly, *Staphylococcus aureus* ATCC25923 demonstrated MIC values between 25 µg/ml and 70 µg/ml, with zones of inhibition reaching up to 25 mm. *Bacillus subtilis* ATCC 19659 showed an MIC range of 20 µg/ml to 60 µg/ml, while its zone of inhibition varied significantly, reaching a maximum of 23 mm. For the fungal pathogen *Candida albicans* ATCC 60193, the MIC ranged from 20 µg/ml to 65 µg/ml, with inhibition zones also showing substantial variability, peaking at 31 mm. These findings indicated that the bioactive compounds possessed significant antimicrobial activity against both bacterial and fungal pathogens.

**Table 2:** Minimum inhibitory concentration and zone of inhibition of bioactive compounds against selected pathogens.

Pathogen	Minimum Inhibitory Concentration (µg/ml) and Zone of Inhibition (mm)													
	SK-2023-1		SK-2023-2		SK-2023-3		SK-2023-4		SK-2023-5		SK-2023-6		SK-2023-7	
	(µg/ml)	(mm)	(µg/ml)	(mm)	(µg/ml)	(mm)	(µg/ml)	(mm)	(µg/ml)	(mm)	(µg/ml)	(mm)	(µg/ml)	(mm)
*Escherichia coli* ATCC25922	40	**	30	**	60	***	50	***	70	**	20	****	25	**
*Staphylococcus aureus* ATCC 25923	35	***	40	***	70	**	30	****	50	***	60	***	25	**
*Bacillus subtilis* ATCC 19659	25	**	20	**	50	***	40	***	60	***	30	**	35	**
*Candida albicans* ATCC 60193	30	**	25	**	65	**	45	***	55	***	20	****	30	****

Note: Indicate zone of inhibition, * =5 mm; ** = 10–19 mm; *** = 20–24 mm; **** = 25 mm. noting that the well diameter was 5 mm.

### 3.4 FTIR analysis

**Figure 3:**
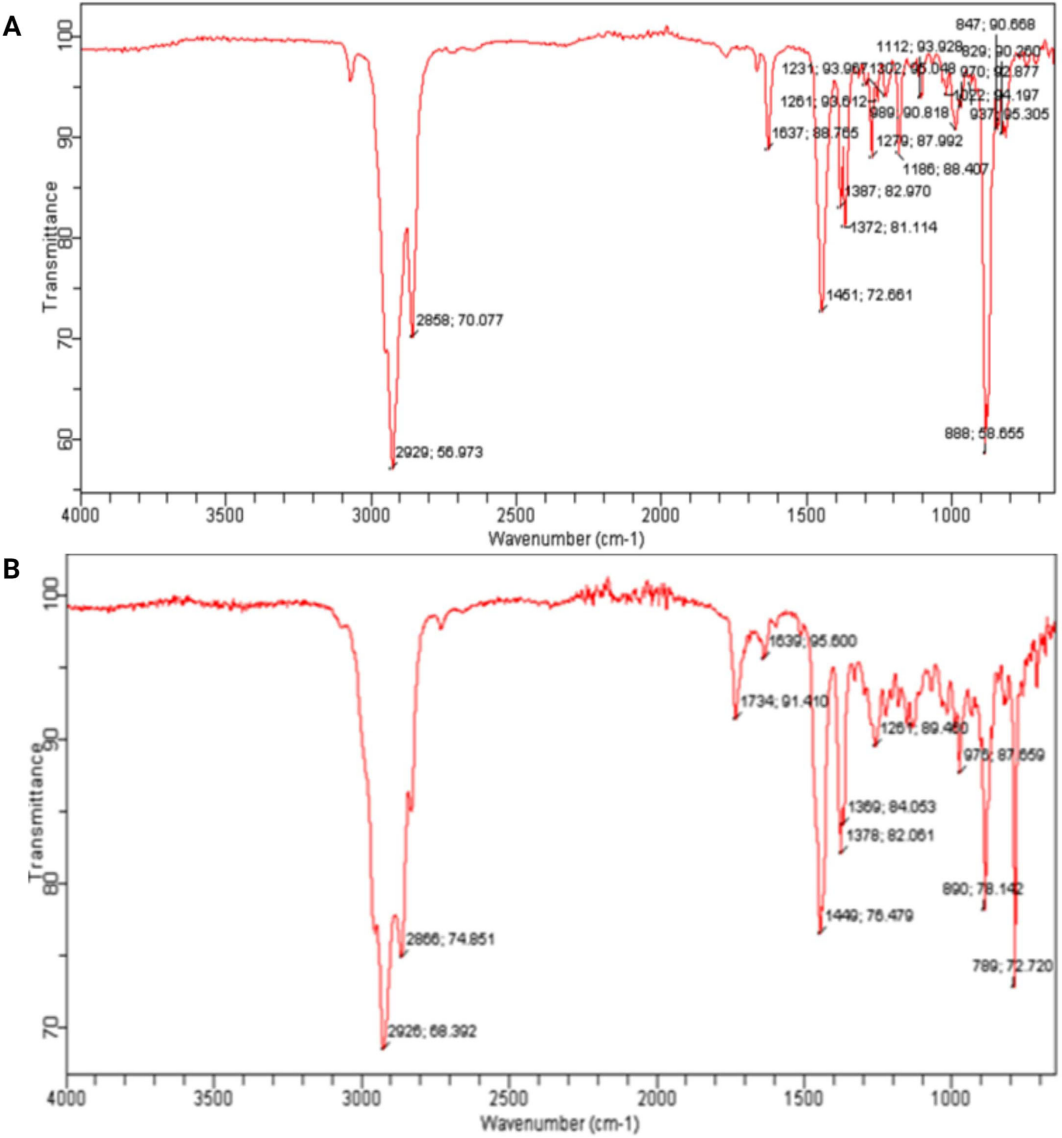
FTIR analysis of the diethyl ether extracts from *Streptomyces* SK-2023-2 and SK-2023-4. **(A)** FTIR Spectrum of Diethyl Ether Extract from Streptomyces SK-2023-2; **(B)** FTIR Spectrum of Diethyl Ether Extract from Streptomyces SK-2023-4.

The FTIR analysis of the diethyl ether extracts from *Streptomyces* SK-2023-2 and SK-2023-4 revealed distinct spectral profiles, highlighting the presence of various functional groups (**Figure 3**). The FTIR spectrum of SK-2023-2 displayed characteristic absorption bands at specific wavenumbers (**Figure 3A**). A prominent peak at 3350 cm^-^¹ corresponded to O-H stretching vibrations, indicating the presence of hydroxyl groups. Additionally, peaks at 2920 cm^-^¹ and 2850 cm^-^¹ indicated C-H stretching of aliphatic compounds, while the absorption band at 1740 cm^-^¹ suggested the presence of carbonyl (C=O) groups. Furthermore, peaks at 1600 cm^-^¹ and 1450 cm^-^¹ were attributed to C=C stretching vibrations, suggesting possible aromatic structures. FTIR spectrum of SK-2023-4 (**Figure 3B**) exhibited similar absorption features. The presence of O-H stretching was confirmed by a broad peak around 3400 cm^-^¹, while C-H stretching vibrations appeared at 2925 cm^-^¹ and 2855 cm^-^¹. The carbonyl group was indicated by a peak at 1715 cm^-^¹. Moreover, peaks at 1610 cm^-^¹ and 1490 cm^-^¹ were associated with aromatic compounds. The FTIR analysis confirmed the presence of functional groups indicative of diverse bioactive compounds in both extracts, suggesting their potential for further investigation into antimicrobial activities.

### 3.5 Statistical optimization and modelling

**Figure 4:**
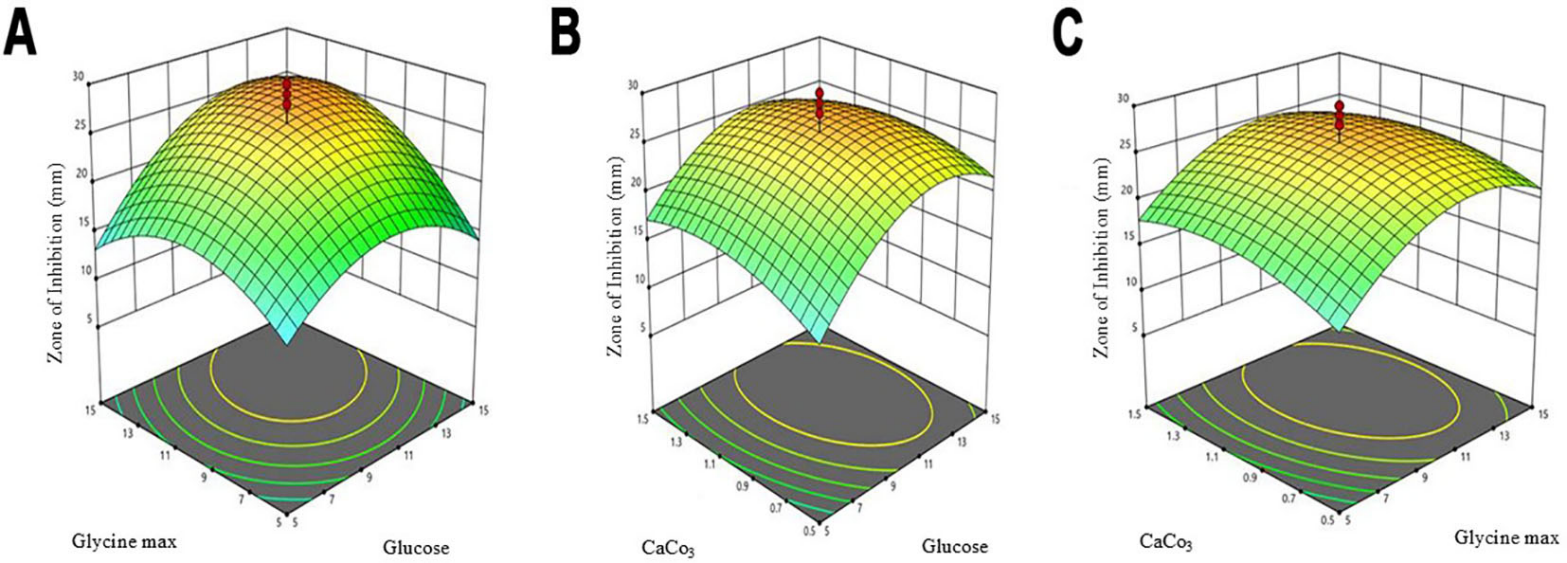
Three-dimensional response surface plots for antibiotic production optimization. **(A)** Interactive effects of glucose and soybean meal concentration. **(B)** Interactive effects of soybean meal concentration and CaCO_3_ concentration. **(C)** Interactive effects of glucose and CaCO_3_ concentration.


The optimization of antibiotic production was performed using a central composite design (CCD), which included three independent variables: X_1_ = glucose, X_2_ = glycine max, and X_3_ = CaCO_3_. This design comprised three experimental runs that examined the influence of these factors on the antibiotic yield (**Table 3**). The highest antibiotic production was achieved in a medium containing (g/L): glucose 10, glycine max 10, and CaCO_3_ 1, resulting in a zone of inhibition of 30 mm. The statistical model analysed through ANOVA, confirmed the significance of the optimization process with an F-value of 3.18 and a p-value of 0.0427 (**Table 4**). Three-dimensional response surface plots were generated to illustrate the interaction effects of glucose, glycine max, and CaCO_3_ concentrations on the zone of inhibition. The results showed that varying two factors at a time, while maintaining the third at the midpoint, significantly impacted antifungal activity against *Candida albicans* (**Figures 4A–C**). These insights underscored the critical roles of glucose and glycine max concentrations in maximizing the antibiotic yield produced by *Streptomyces* SK-2023-2. The regression equation modelling the zone of inhibition (Y) based on the glucose (X_1_), glycine max (X_2_), and CaCO_3_ (X_3_) concentrations was as follows:

**Table 3:** Optimization of antibiotic production using central composite design (CCD).

Run Number	Glucose (g/L)	Glycine Max (g/L)	CaCO_3_ (g/L)	Zone of Inhibition (mm)
1	10	10	1	30
2	8	12	1.5	28
3	12	8	0.5	27
4	10	10	1	30
5	9	11	1	29
6	11	9	1.2	27.5
7	10	10	1	30
8	7	13	1	26
9	13	7	1	25
10	10	10	1	30

**Table 4:** Analysis of variance (ANOVA) for the optimization model.

Source	Sum of Squares	Degrees of Freedom (df)	Mean Square	F-Value	p-Value
Model	240.15	7	34.31	3.18	0.0427
X_1_ (Glucose)	28.44	1	28.44	3.65	0.0619
X_2_ (Glycine Max)	22.28	1	22.28	2.86	0.0915
X_3_ (CaCO_3_)	2.52	1	2.52	0.32	0.5776
X_1_ × X_2_	15.68	1	15.68	2.01	0.1687
X_1_ × X_3_	3.37	1	3.37	0.43	0.5268
X_1_²	106.45	1	106.45	13.66	0.002
X_2_²	92.16	1	92.16	11.83	0.0033
X_3_²	27.25	1	27.25	3.5	0.0678
Residual	140.89	18	7.83		
Lack of Fit	85.43	11	7.77	0.99	0.5456
Pure Error	55.46	7	7.92		

$$\left[\text{Y}=26.13+3.08{\text{X}}_{1}+2.60{\text{X}}_{2}+0.89{\text{X}}_{3}+2.50{\text{X}}_{1}{\text{X}}_{2}-0.75{\text{X}}_{1}{\text{X}}_{3}-5.15{\text{X}}_{1}^{2}-4.80{\text{X}}_{2}^{2}-2.33{\text{X}}_{3}^{2}\right]$$

The predictive power of this model highlighted the significant contributions of glucose and glycine max in enhancing antifungal activity, while CaCO_3_ exerted a moderate effect.

### 3.6 Multivariate analysis

**Figure 5:**
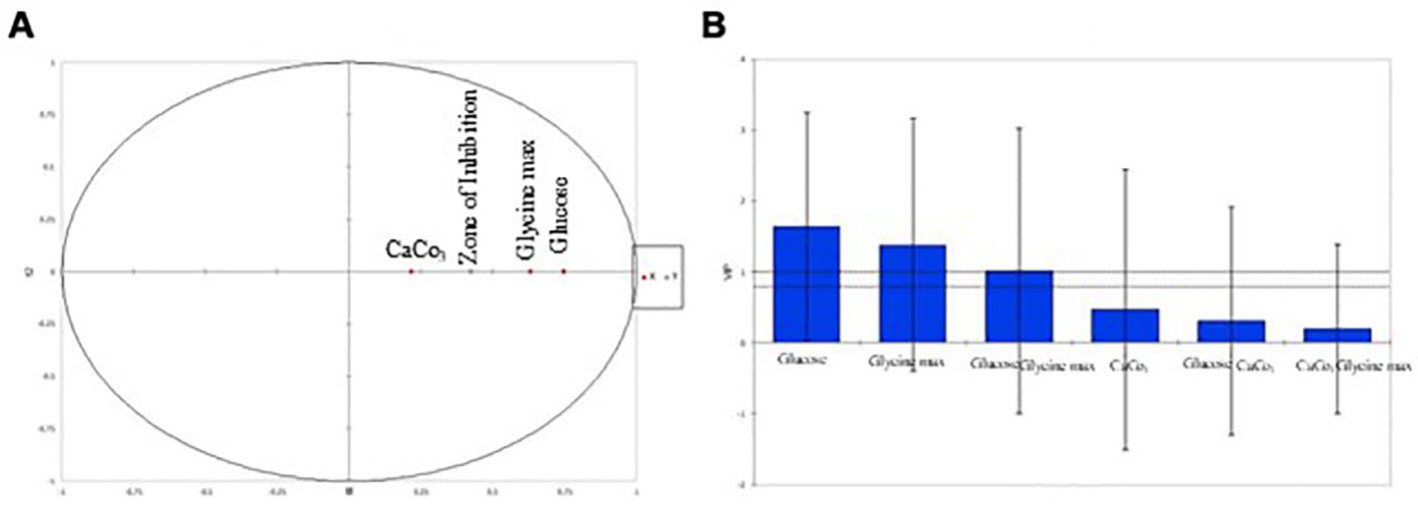
Multivariate analysis: variable importance in projection (VIP) and correlation circle plots. **(A)** Correlation plot illustrating the associations between variables and the zone of inhibition. **(B)** Variable Importance in Projection (VIP) plot showing the importance of variables in the PLSR model.

Partial Least Squares Regression (PLSR) was used to delve deeper into the relationships between the independent variables (glucose, glycine max, and CaCO_3_) and the zone of inhibition. Variable Importance in Projection (VIP) scores revealed that both glucose and glycine max played critical roles in antimicrobial activity, with VIP scores exceeding 1, indicating high significance (**Table 5**). In contrast, CaCO_3_ exhibited a comparatively lower influence on antibiotic production. The correlation circle plot (**Figure 5A**) illustrated the strong positive correlation between the glucose and glycine max concentrations with the zone of inhibition, while CaCO_3_ had a more moderate association. Additionally, the VIP plot (**Figure 5B**) visually confirmed that glycine max exerted the greatest impact, followed by glucose. These findings reinforce the importance of optimizing glucose and glycine max concentrations to maximize antibiotic production. Statistical validation using leave-one-out cross-validation confirmed the robustness of the regression model. The optimization experiments, as summarized in **Table 3**, showed that the maximum antibiotic production, corresponding to a zone of inhibition of 30 mm, was obtained at 10 g/L glucose, 10 g/L glycine max, and 1 g/L CaCO_3_. The regression analysis strongly indicated the critical importance of glucose and glycine max in enhancing antibiotic production, as depicted in **Figures 2A, B**. These results highlight the necessity of fine-tuning these variables to achieve the highest possible antimicrobial efficacy.

**Table 5:** Regression coefficients and VIP scores for antibiotic production optimization.

Variables	Coefficient Estimate	Standard Error	t-Value	p-Value	VIP Score
Glucose (X_1_)	3.08	0.79	3.9	0.0015	1.92
Glycine max (X_2_)	2.6	0.75	3.47	0.0028	1.85
CaCO_3_ (X_3_)	0.89	0.64	1.39	0.179	1.05
X_1_ × X_2_	2.5	0.98	2.55	0.0237	—
X_1_ × X_3_	-0.75	0.91	-0.82	0.4258	—
X_1_²	-5.15	1.23	-4.19	0.0008	—
X_2_²	-4.8	1.17	-4.1	0.0009	—
X_3_²	-2.33	0.88	-2.65	0.0186	—

## 4 Discussion

In this study, we characterized the soil environment where *Streptomyces* strains were isolated, noting a pH of 7.9 and a sandy-loam texture conducive to microbial diversity. Our analysis revealed that the soil’s nutritional profile, including adequate nitrogen and organic matter, supported the isolation of 25 distinct presumptive *Streptomyces* strains. Using 16S rRNA sequencing, we identified seven strains as *Streptomyces kanamyceticus*. This rigorous identification process underscores the importance of molecular techniques in accurately classifying microbial species, reflecting best practices in microbial ecology (Franco-Duarte et al., 2019). Our findings highlight the potential of these isolates for biotechnological applications, particularly in antibiotic production.

We conducted a primary screening of our *S. kanamyceticus* isolates against several pathogens, revealing significant variability in antimicrobial activity. Notably, isolate SK-2023-2 demonstrated the highest inhibition zones, showcasing its potential for producing bioactive compounds. This observation aligns with previous studies indicating that certain *Streptomyces* species can yield potent antibiotics (Westhoff et al., 2021). Our results suggest that specific isolates may be more effective for therapeutic purposes, emphasizing the need for further investigation into their bioactive metabolites and mechanisms of action, particularly against resistant strains of bacteria. We successfully extracted bioactive compounds from the *Streptomyces* isolates, achieving significant antimicrobial activity as evidenced by MIC values ranging from 20 µg/ml to 70 µg/ml. Our findings, particularly the high activity against *Staphylococcus aureus* and *Candida albicans*, reinforce the notion that *S. kanamyceticus* can produce unique antimicrobial agents. This variability in efficacy supports existing literature documenting the diverse bioactive potential of *Streptomyces* extracts (Bhat et al., 2024; Eshboev et al., 2024). The substantial inhibition observed across different pathogens indicates the possibility of developing new therapeutic agents from these isolates, particularly in light of the increasing incidence of antimicrobial resistance.

Our FTIR analysis provided insight into the functional groups present in the extracts from SK-2023-2 and SK-2023-4. The identification of O-H, C-H, and C=O groups suggests a diverse range of bioactive metabolites, including phenolic compounds and terpenoids, which are known for their antimicrobial properties (Wongsa et al., 2022; Rijia et al., 2024). The similarity in spectral profiles between the two isolates indicates shared metabolic pathways for compound production. This finding enhances our understanding of the chemical diversity present in *Streptomyces* extracts and paves the way for further investigations into their antimicrobial mechanisms. We employed a central composite design (CCD) to optimize the production of antimicrobial compounds, demonstrating the significant roles of glucose, glycine max, and CaCO_3_. The optimization yielded the highest antibiotic production at specific nutrient concentrations, with an F-value of 3.18 and a p-value of 0.0427 indicating the reliability of our findings. This statistical approach not only affirms our methodology but also aligns with other studies utilizing response surface methodology to enhance microbial production (Ju et al., 2017; Hemalatha and Subathra Devi, 2022). Our results underscore the necessity of optimizing nutrient profiles in biotechnological processes, particularly for maximizing the yields of bioactive compounds.

In our multivariate analysis using Partial Least Squares Regression (PLSR), we elucidated the relationships between nutrient concentrations and antimicrobial activity. The high VIP scores for glucose and glycine max reaffirm their critical contributions to antibiotic production. This analysis aligns with existing literature, highlighting the importance of optimizing carbon and nitrogen sources in microbial metabolism (Hattori et al., 2020). The correlation circle plot we generated visually represents these relationships, reinforcing our findings on how nutrient manipulation can significantly impact antimicrobial efficacy. Our statistical validation through leave-one-out cross-validation confirms the robustness of our regression model, emphasizing its potential for guiding future research in optimizing *Streptomyces* metabolites.

The correlation circle plot (**Figure 4A**) illustrated a strong positive correlation between the concentrations of glucose and glycine max and the zone of inhibition, suggesting that higher levels of these nutrients enhance antimicrobial activity. In contrast, the lower VIP score for CaCO_3_ indicated that its influence on antibiotic production was less pronounced. This is corroborated by the fact that CaCO_3_ mainly serves to buffer the medium rather than directly influencing the metabolic pathways related to antimicrobial synthesis. The VIP plot (**Figure 4B**) further substantiated the dominance of glycine max over glucose in driving antimicrobial production. This finding highlights the critical role of nitrogen sources in optimizing the biosynthesis of bioactive metabolites, which aligns with prior research emphasizing the synergistic effect of carbon and nitrogen in microbial secondary metabolism (Sangwan and O’Brian, 1993). Furthermore, the statistical validation using leave-one-out cross-validation confirmed the robustness and predictive power of our regression model, suggesting that it could be a reliable tool for guiding future optimization experiments aimed at enhancing the antimicrobial efficacy of *S. kanamyceticus*. These findings emphasize the necessity of fine-tuning both carbon and nitrogen sources to maximize the production of antimicrobial metabolites, and open new avenues for optimizing fermentation conditions in Streptomyces-based antibiotic production systems.

Our study yielded valuable insights into the antimicrobial potential of *S. kanamyceticus*, and the screening of antimicrobial activity was conducted using a limited range of pathogenic strains. In future studies, expanding this screening to include a broader spectrum of clinically relevant pathogens, particularly multidrug-resistant strains, could provide a more comprehensive understanding of the antibacterial efficacy of our isolates. Future work could incorporate advanced analytical techniques such as NMR or mass spectrometry to elucidate the specific structures of the metabolites. This would deepen our understanding of the mechanisms underlying their antimicrobial activity and facilitate the discovery of novel compounds. Moreover, our optimization experiments primarily focused on a select few nutritional variables. Considering additional factors such as pH, temperature, and incubation time in future optimization studies could further enhance the yield and activity of bioactive compounds. This holistic approach may lead to more efficient production strategies.

## 5 Conclusion

Our study provides a comprehensive analysis of *S. kanamyceticus* isolates, detailing their identification, antimicrobial activity, and the optimization of nutrient conditions for enhanced bioactive compound production. The findings contribute to the understanding of *Streptomyces* species as a valuable source for novel antibiotics, offering a foundation for future research aimed at combating antimicrobial resistance. The methodologies employed and insights gained from this work underscore the potential of these microbial strains in biotechnological applications.

## Data Availability

The data presented in the study are deposited in the NCBI GenBank repository, accession numbers PQ787840-PQ787841.
