# Supplementary material for: Optimizing the production and efficacy of antimicrobial bioactive compounds from Streptomyces kanamyceticus in combating multi-drug-resistant pathogens
Source: Front Cell Infect Microbiol. 2025 Jan 6;14:1500440. doi: 10.3389/fcimb.2024.1500440 (PMC11743287; doi:10.3389/fcimb.2024.1500440)
Supplement: Supplementary file 1 [file Table1.docx]

Supplementary File

Table 1 Soil Characterization and Chemical Analysis Data

| **Parameter** | **Value** |
| --- | --- |
| Soil pH | 7.9 (alkaline) |
| Electrical Conductivity (EC) | 0.15 ds/m |
| Soil Texture | Sandy Loam |
| Clay Content | 20% |
| Total Nitrogen | 0.17% |
| Organic Matter | 2.98% |
| **Exchangeable Cations** |  |
| Potassium (K) | 1.50% |
| Magnesium (Mg) | 1% |
| Aluminium (Al) | 6% |
| Calcium (Ca) | 29% |
| Silicon (Si) | 3.50% |
